# Antiplasmodial potential and quantification of aloin and aloe-emodin in *Aloe vera* collected from different climatic regions of India

**DOI:** 10.1186/s12906-017-1883-0

**Published:** 2017-07-17

**Authors:** Sandeep Kumar, Manila Yadav, Amita Yadav, Pooja Rohilla, Jaya Parkash Yadav

**Affiliations:** Department of Genetics, Maharshi Dayanand University, Rohtak, Haryana -124001 India

**Keywords:** Quantitative analysis, *Aloe vera*, Anthraquinone, Climatic zone, Antiplasmodial

## Abstract

**Background:**

In this study, *Aloe vera* samples were collected from different climatic regions of India. Quantitative HPTLC (high performance thin layer chromatography) analysis of important anthraquinones aloin and aloe-emodin and antiplasmodial activity of crude aqueous extracts was done to estimate the effects of these constituents on antiplasmodial potential of the plant.

**Methods:**

HPTLC system equipped with a sample applicator Linomat V with CAMAG sample syringe, twin rough plate development chamber (20 x 10 cm), TLC Scanner 3 and integration software WINCATS 1.4.8 was used for analysis of aloin and aloe-emodin amount. The antiplasmodial activity of plant extracts was assessed against a chloroquine (CQ) sensitive strain of *P. falciparum* (MRC-2). Minimum Inhibitory Concentration (MIC) of aqueous extracts of selected samples was determined according to the World Health Organization (WHO) recommended method that was based on assessing the inhibition of schizont maturation in a 96-well microtitre plate. EC (effective concentration) values of different samples were observed to predict antiplasmodial potential of the plant in terms of their climatic zones.

**Results:**

A maximum quantity of aloin and aloe-emodin i.e. 0.45 and 0.27 mg/g respectively was observed from the 12 samples of *Aloe vera*. The inhibited parasite growth with EC_50_ values ranging from 0.289 to 1056 μg/ml. The antiplasmodial EC_50_ value of positive control Chloroquine was observed 0.034 μg/ml and EC_50_ values showed by aloin and aloe-emodin was 67 μg/ml and 22 μg/ml respectively. A positive correlation was reported between aloin and aloe-emodin. Antiplasmodial activity was increased with increase in the concentration of aloin and aloe-emodin. The quantity of aloin and aloe-emodin was decreased with rise in temperature hence it was negatively correlated with temperature.

**Conclusions:**

The extracts of *Aloe vera* collected from colder climatic regions showed good antiplasmodial activity and also showed the presence of higher amount of aloin and aloe-emodin in comparison to collected from warmer climatic sites. Study showed significant correlation between quantities of both the anthraquinones used as marker compounds and EC_50_ values of the different *Aloe vera* extracts. Although, both the anthraquinones showed less antiplasmodial potential in comparison to crude extracts of different *Aloe vera* samples. Diverse climatic factors affect the quantity of tested compounds and antiplasmodial potential of the plant in different *Aloe vera* samples.

## Background

Throughout the history of mankind, malaria has been one of the major causes of human illness and death. More than 800,000 deaths occur every year; the vast majority being children under the age of five. Thus this highly infectious disease has a global impact. Malaria is a parasitic disease widespread in tropical and subtropical regions of the world [1, 2]. It is endemic particularly in regions of Africa, Asia and South America. India’s extensive geography and diverse climate supports ideal environments for sustaining malarial parasites and their vectors [3]. Malaria can be diagnosed easily on morphological basis at different stages of parasite in human blood; with the exception of *P. falciparum.* [4]. *P. falciparum* is the most severe strain of the malaria due to highest human deaths and resistant to standard antimalarial drugs. [5]. The WHO has recommended artemisinin-based combination therapy (ACT) as the first line treatment for multidrug resistant malaria caused by *P. falciparum* in different parts of the word [6]. Recent studies have reported that *P. falciparum* has developed resistance to many of available antimalarial drugs [7–9].

Malaria has become a leading cause of morbidity and mortality mainly due to its prevalence in poor resource countries; where the therapy is unaffordable due to non-availability of oral administered drugs [10]. As antimalarial drug resistance is undermining the effective treatment of the disease; there is a critical need for effective, safe, and affordable antimalarial agents. Herbal medicine occupies a pivotal role in treating infectious diseases since onset of mankind. It is estimated that about 40% of all medicines is either natural products or their semi-synthetic derivatives [11].

Natural products may offer relatively cheap alternative treatment opportunities for malaria patients due to vast metabolic diversity [12–14]. Currently used antimalarial drugs such as quinine and artemisinin were both isolated from plants *Cinchona officinalis* and *Artemisia annua* respectively. Consequently, it has been established that plants have potential as sources for antimalarial drugs. The diverse climates of India flourish huge diversity of medicinal plants and use of plants for treating ailments tracks back to ancient Indian [15].

Quality evaluation and pharmacological standardization of herbal preparation is a fundamental requirement of industry for commercial production. According to WHO guidelines, an herbal product needs to be standardized with respect to safety before releasing it into the market [16]. HPTLC (high performance thin layer chromatography) is an inexpensive method for separation, qualitative identification, or semi-quantitative analysis of samples and it can be used to solve many qualitative and quantitative analytical problems in a wide range of fields; including medicine, pharmaceuticals, chemistry, biochemistry, food analysis, toxicology and environmental analysis [17].

*Aloe vera* (syn.: *Aloe barbadensis* Miller) is the most commercialized *Aloe* species belonging to the Xanthorrhoeaceae family [18, 19]. There are many natural medicinal herbs, but *Aloe vera* possesses a vast array of healing benefits. Owing to its multipurpose utility, *Aloe* has been introduced into cultivation as a household plant. It has been in use since ages as folk medicine. *Aloe vera* is a rich source of over 200 naturally occurring nutrients which contain water soluble and fat soluble vitamins, minerals, enzymes, polysaccharides, phenolic compounds and organic acids [20]. Its secondary metabolites have multiple properties such as anti-inflammatory, antibacterial, antioxidant, immune boosting, anticancer, antiageing, sunburn relief and antidiabetic potentials [21–23]. Several traditional uses also have been reported such as burn injury, eczema, cosmetics, inflammation, and fever [24]. *Aloe* juice mixed with water and honey is used as an effective antimalarial cure in Yemen [25].

Recently *Aloe vera* was reported to be used against malaria parasite with the highest frequency in a documentation report on medicinal plants used by the local communities of western Uganda [26]. Van Zyl and Viljoen [27] screened the main constituents of 34 *Aloe* species for antiplasmodial activity using the titrated hypoxanthine incorporation assay. They have observed that methanol extracts possessed antiplasmodial activity against *Plasmodium falciparum* strain at concentration ranged from 32 to 77 μg ml^−1^ where 50% of the parasite growth was inhibited (IC_50_ value) [27]. The Aloe species of *A. secundiflora* and *A. lateritia* were also used for treating malaria and related symptoms [28].

Geographical conditions are the main factors that ultimately affect the phytoconstituents and medicinal properties of a plant. India has six major climatic zones: Highland, semi-arid, arid, tropical wet, tropical wet and dry, and humid subtropical climate. *Aloe vera* grows all over the India, wildly in Maharashtra and Tamil Nadu states where as Andhra Pradesh, Gujarat and Rajasthan states are known for its cultivation. [29]. So, keeping in view of the importance of *Aloe vera* plant the present work is an attempt to evaluate the antiplasmodial activity and phytochemical standardization of *Aloe vera* aqueous extracts with HPTLC; collected from different climatic regions of India to elaborate the effect of climatic conditions on phytochemical diversity and activity of samples.

## Methods

### Plant collection

**Fig. 1:**
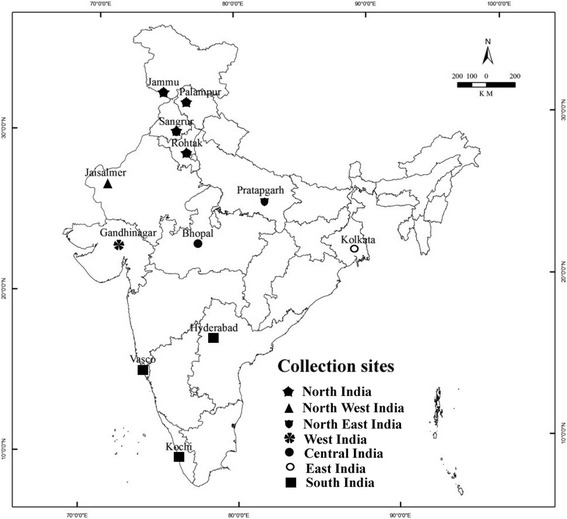
Map showing *Aloe vera* sample collection sites from different places of India

Samples were collected from 12 different sites of north to south India covering 6 agro-climatic zones of India. Each zone had 2 sites (Fig. 1). Geographical locations of collection sites along with their average temperature and rainfall are depicted in Table 1 **.** Samples were collected in the months of January and February 2013. Healthy leaves of *Aloe vera* were collected from individual wild plants at each location. The plant material was identified and authenticated by comparing the herbarium specimen (MDU-6803) available in Department of Genetics, M. D. University, Rohtak (India). Tissues were placed in sterile plastic bags. All samples were brought to the laboratory in an ice box and processed further.

**Table 1:** Geographical parameters, crude extract yield, yield of aloin and aloe-emodin and antiplasmodial EC_50_ values of *Aloe vera* aqueous extracts collected from different sites of India

Sr. No.	Agro-climatic zones	Collection sites	Place of collection	Average Temp. (°C).	Average Rainfall (mm).	Crude Extract Yield (g/100 g)	Aloin Yield out of crude extract		Aloe-emodin Yield out of crude extract		EC_50_ (μg/ml) for antiplasmodial activity
							(mg/g)	% yield	(mg/g)	% yield	
1	Highland	Jammu and Kashmir [J&K]	Jammu	13.5	1011	3.7	0.45 ± 0.061	1.21	0.23 ± 0.054	0.62	0.441 ± 0.093
2		Himachal Pradesh [H.P.]	Palampur	13.5	1251	3.5	0.45 ± 0.043	1.28	0.21 ± 0.008	0.60	0.289 ± 0.032
3	Semi-arid	Punjab	Sangrur	25	649	3.9	0.32 ± 0.050	0.82	0.29 ± 0.066	0.74	0.472 ± 0.012
4		Haryana	Rohtak	27	617	4.1	0.39 ± 0.022	0.95	0.11 ± 0.037	0.27	0.407 ± 0.085
5	Arid	Rajasthan	Jaisalmer	28.5	209.5	3.5	0.37 ± 0.034	1.06	0.27 ± 0.029	0.77	0.980 ± 0.007
6		Gujarat	Gandhinagar	27.5	1107	3.8	0.21 ± 0.067	0.55	0.13 ± 0.043	0.34	0.621 ± 0.036
7	Humid Subtropical	Uttar Pradesh [U.P.]	Pratapgarh	25.5	904	3.9	0.33 ± 0.041	0.85	Peak not detected	**---**	0.299 ± 0.059
8		Madhya Pradesh [MP]	Bhopal	25.5	1146	3.0	0.22 ± 0.074	0.73	0.19 ± 0.060	0.63	0.689 ± 0.027
9	Tropical wet & dry	West Bengal [W.B.]	Kolkata	27	1582	3.4	0.17 ± 0.037	0.50	0.27 ± 0.036	0.79	0.417 ± 0.041
10		Telangana	Hyderabad	26.5	812.5	3.7	Peak not detected	**---**	Peak not detected	**---**	1056 ± 0.033
11	Tropical wet	Kerala	Kochi	28	3005	3.6	Peak not detected	**---**	0.09 ± 0.042	0.25	0.558 ± 0.047
12		Goa	Vasco	27.5	3055	3.3	0.15 ± 0.033	0.45	0.20 ± 0.040	0.60	0.663 ± 0.029

Positive control Chloroquine (CQ) value = 0.034 ± 0.027 μg/ml; aloin = 67 ± 0.046 μg/ml; aloe-emodin = 22 ± 0.063 μg/ml

### Extract preparation

The samples were first washed with tap water and then surface sterilised in 10% sodium hypochlorite to prevent the contamination of any microbes [30]. They were thoroughly rinsed with sterile distilled water. The plant samples were shade dried followed by oven drying (50 °C) and milled in an electrical blender. Aqueous extracts of different accessions were prepared by cold percolation method. The extracts were pooled, and the solvent was evaporated using a rotary evaporator under reduced pressure at 40 °C. The crude extracts thus obtained were kept at 4 °C for antiplasmodial assay.

### HPTLC analysis

A CAMAG (Muttenz, Switzerland) HPTLC system equipped with a sample applicator Linomat V with CAMAG sample syringe, 100 μl, twin rough plate development chamber (20 × 10 cm), TLC Scanner 3 and integration software WINCATS 1.4.8 was used. CAMAG TLC visualiser (Professional photo documentation system) was used for capturing the coloured images of the developed TLC plate and other documentation purposes. An aluminium supported HPTLC plate (20 × 10 cm) pre-coated with silica gel 60F 254 (E. Merck) was used as an adsorbent. CAMAG TLC plate heater was used to pre-activation of the HPTLC plates and for drying the developed plates. The experimental conditions maintained were temperature 25 ± 2 °C, relative humidity 40%.

TLC method was used for estimation of aloin and aloe-emodin content in different samples of *Aloe vera* using standard berberine as biomarker*.* HPTLC precoated plates Silica Gel Merck 60F254 was used as a stationary phase.

*Aloe vera* aqueous extract of different samples was made to attain the final concentration of 1000 μg/ml. A 1000 μg/ml solution of aloin and aloe-emodin reference standard was prepared in water as a stock solution. These solutions were used for further HPTLC analysis as per the developed protocol. Ethyl acetate, methanol and water in the ratio of 10:2:1 (*v*/v/v) were used as a mobile phase.

The TLC plate (10 × 10 cm) was pre-activated on TLC plate heater at 60 °C for 30 min. For co-chromatography with aloin and emodin, 10 μl of sample solution of aqueous extract along with the standard was applied on a TLC plate and the plate was developed in Ethyl acetate, methanol and water in the ratio of 10:2:1 (*v*/v/v) solvent system to a distance of 8 cm. The plates were dried at 120 °C temperature for 5 min using TLC plate heater. The plate was then kept inside TLC visualiser for resolving the coloured bands and for plate photo documentation. The Retardation factor (R_f_) values and colour of the resolved bands were noted.

### HPTLC densitometry analysis

#### Preparation of sample & standard solution

Sample solution (aqueous extract; 1000 μg/ml) and standard solution (1000 μg/ml) described under the previous section was used for quantification of aloin and emodin.

#### Preparation of calibration curve of & quantification of aloin and aloe-emodin

The different volume of standard stock solution 2, 4, 6, 8, 10 and 12 μl were spotted on HPTLC plate (20 × 10 cm) in order to deliver concentration of 4, 6, 8, 10 and 12 μg/spot of aloin and aloe-emodin respectively using vial I and vial II respectively followed by spotting of 4 μl of sample stock solution of 12 samples. Samples were applied as bands 4 mm wide, 12 mm apart, by CAMAG Linomat V applicator using 100 μl sample syringe with a constant application rate of 150 nLs^−1^. After sample application plates were developed in a developing chamber pre-saturated with the mobile phase (20 ml) Ethyl acetate, methanol and water in the ratio of 10:2:1 (*v*/v/v) for 20 min. The plate was developed in CAMAG horizontal twin tough glass developing chamber (20 × 10 cm) at the room temperature up to 8 cm (80% of the total plate size). Ascending mode was used for the development of thin layer chromatography.

After development, plates were dried on CAMAG TLC plate heater at 120 °C temperature for 5 min. The plate was then observed under UV and fluorescence reflectance mode in CAMAG TLC visualiser photo documentation system at wavelength of 254 and 366 nm respectively. The coloured images of the developed plates were documented using WINCATS 1.4.8 software at both wavelengths 254 and 366 nm**.** The developed plate was then scanned at 366 nm using CAMAG TLC densitometric scanner 3 integrated with WINCATS 1.4.8 software.

The calibration curve was prepared using standard concentration range of 4–12 μg/spot. Each concentration peak area was plotted against the concentration of aloin and aloe-emodin respectively spotted in densitometric analysis within the integrated software. Quantification of aloin and aloe-emodin was done as the peak areas of sample spots were recorded and the amount of aloin and aloe-emodin were calculated using standard curve.

### Antiplasmodial activity

#### Parasite culture

The antiplasmodial activity of plant extracts was assessed against a chloroquine (CQ)-sensitive strain of *P. falciparum* (MRC-2) obtained from the National Institute of Malaria Research, New Delhi, and maintained in continuous culture according to the methodology described by Trager and Jensen [31]. Parasites were cultivated in group O^+^ human erythrocytes and suspended at a 4% hematocrit in RPMI-1640 medium supplemented with HEPES, NaHCO_3_, 10% O^+^ human serum and neomycin at 37 °C in a controlled gas atmosphere of 2% O_2_, 5% CO_2_ and 93% N_2_ in a CO_2_ incubator (Galaxy 170R, New Brunswick, USA).

#### Minimum inhibitory concentration (MIC)

The antiplasmodial activity of aqueous extracts of selected samples were performed in triplicate in a 96-well microtitre plate, according to the method of WHO that was based on assessing the inhibition of schizont maturation [32]. The culture was synchronised using 5% (*w*/*v*) of sorbitol for 5 min at room temperature to ensure killing of all other stages except rings. It was centrifuged for 5 min at 1500 rpm. The supernatant was discarded, and the pellet was washed two times with incomplete media. Parasitaemia was adjusted to about 1% for the assay by diluting with freshly washed RBCs. The extracts were dissolved in DMSO to obtain the concentrations of 125, 62.5, 31.25, 15.6, 7.8, 3.9 and 1.9 μg/mL. For the positive control wells, parasitized red blood cells were devoid of extracts, whereas only non-parasitized red blood cells were prepared for the negative control wells. Fifty microlitres of blood mixture media was added to each well in the plate and incubated in controlled gas atmosphere of 2% O_2_, 5% CO_2_ and 93% N_2_ at 37 °C for 24–36 h in a CO_2_ incubator.

After incubation, contents of the wells were harvested and stained for 30 min in a 2% Giemsa solution (pH 7.2). The EC_50_ value for the standard drug CQ (Chloroquine) was also recorded. The developed schizonts were counted against the total asexual parasite count of 200.

### Statistical analysis

Effective concentration of the extracts was determined by using software HN-NonLin V1.1. EC_50_ values, indicating the concentration of the extract required to obtain 50% inhibition of parasite growth, were calculated by linear regression analysis with three replicates. Pearson coefficient method was used to exhibit correlation between quantitative phytochemicals analyses and antiplasmodial activity of each extracts. Correlation between aloin, aloe-emodin quantity, EC_50_ values, temperature and rainfall of each collection sites was calculated using SPSS software version 16.

## Results

The yields of crude aqueous extracts alongwith aloin and aloin-emodin have been depicted in Table 1.

### HPTLC analysis

Developed TLC method resolved the aloin at R_f_ value of nearly about 0.80 and aloe-emodin at 0.83 confirming presence of aloin and aloe-emodin in plant extracts visualised by bluish/blackish band parallel to aloin and aloe-emodin spot along with other resolved components in developed TLC plate.

#### HPTLC densitometry analysis

**Fig. 2:**
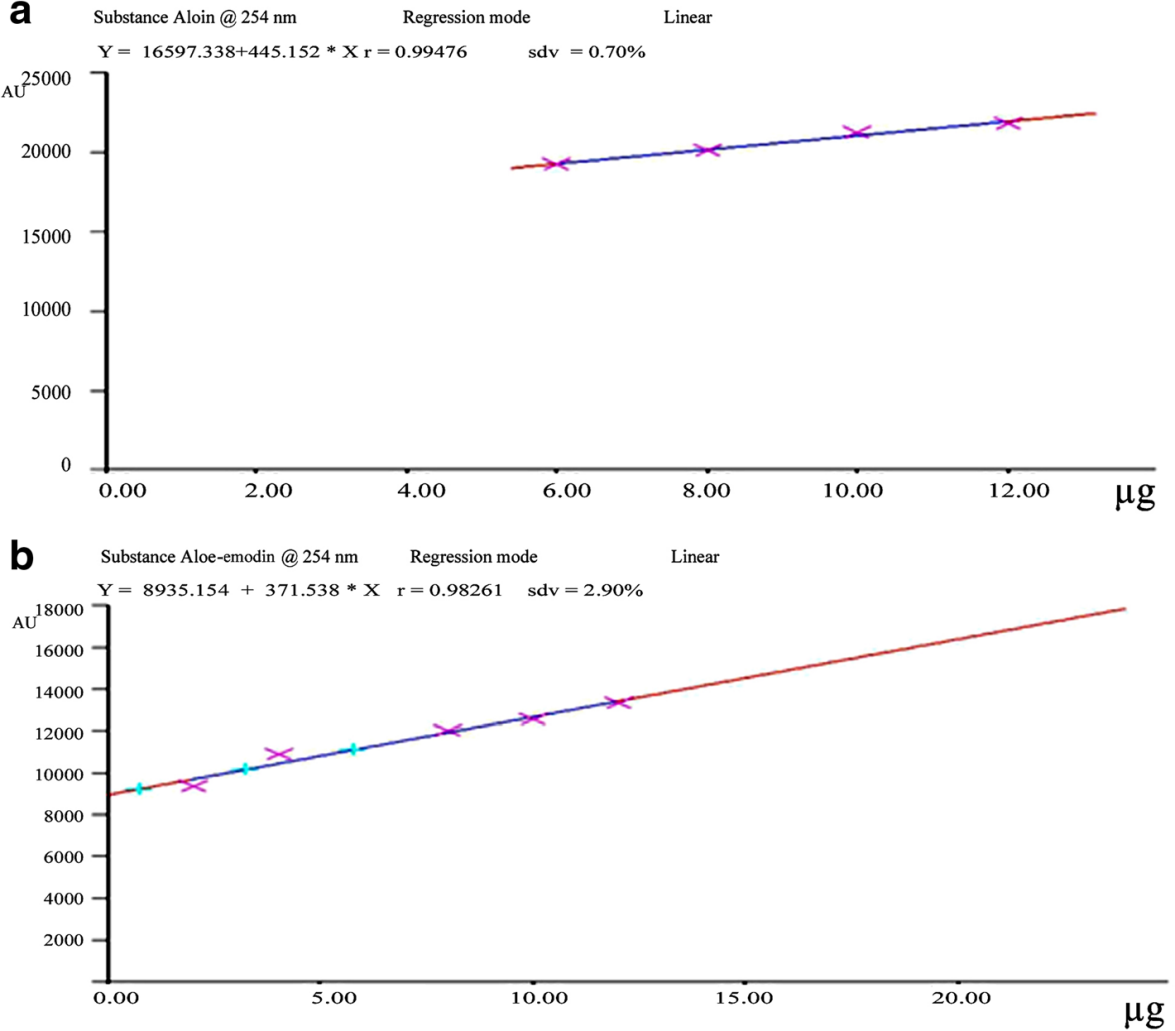
Calibration curve showing linear regression of standard curve of aloin (**a**) and aloe-emodin (**b**)

The spot at R_f_ 0.80 was identified as aloin and at 0.83 was identified as aloe-emodin with the help of chromatogram of the standard compound and HPTLC plate image at 254 nm and 366 nm. The well defined spots were obtained upon complete saturation of the solvent chamber for 30 min. The peak corresponding to aloin and emodin from the sample solution had same retention factor as that from aloin and emodin standard (rf 0.80 and 0.83). From the standard curve of aloin and aloe-emodin quantification of standards were done which revealed the amount of aloin and aloe-emodin present in the samples (Fig. 2). The quantity of aloin ranged from 0.15 to 0.45 mg/g and aloe-emodin from 0.09 to 0.29 mg/g. Percent yield of aloin and aloe-emodin has been calculated from the quantity of crude extract.

The linear regression of standard curve of aloin was determined with R^2^ ± SD = 0.994 ± 0.70%. The linear regression line is y = 445.152× + 18,597.338 (Fig. 2.a). The linear regression of standard curve of aloin-emodin was determined with R^2^ ± SD = 0.982 ± 0.2.90%. The linear regression line is y = 371.539× + 9935.154 (Fig. 2.b).

### Antiplasmodial activity

**Fig. 3:**
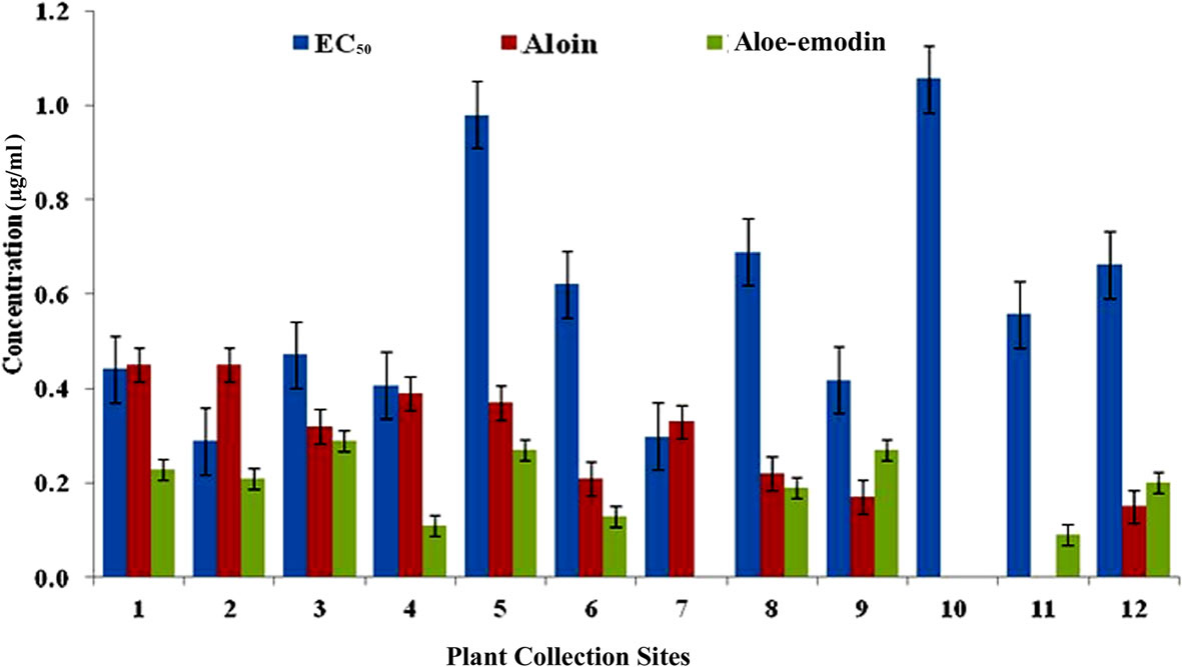
Antiplasmodial EC_50_ values, quantity of aloin and aloe-emodin from crude aqueous extracts of different *Aloe vera* samples

**Fig. 4:**
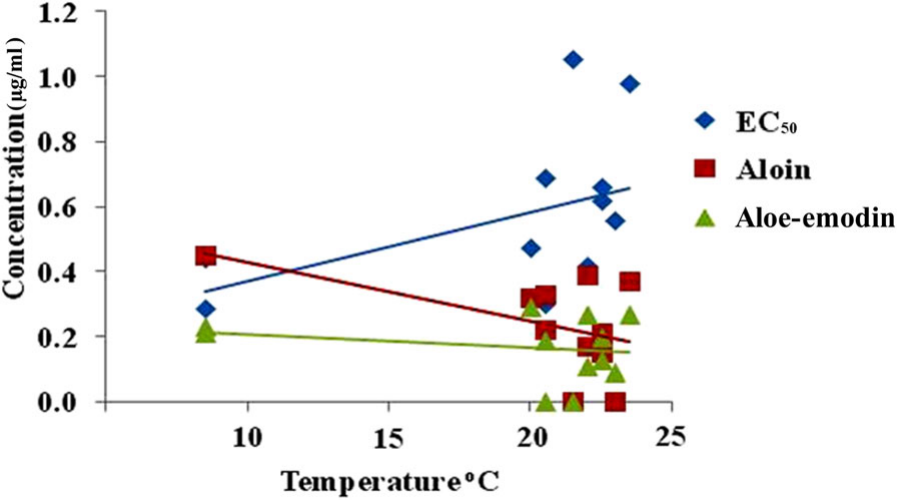
Correlation analysis of temperature with aloin and aloe-emodin quantities and antiplasmodial EC_50_ values of different *Aloe vera* samples

Crude aqueous extracts from different Indian *Aloe vera* samples were evaluated for antiplasmodial activity in terms of EC_50_ values. EC_50_ values for different crude extracts ranged from 0.289 to 1056 μg/ml (Table 1). Himachal Pradesh (0.289 μg/ml) sample showed the lowest EC_50_ value, which was followed by Uttar Pradesh (0.299 μg/ml) sample. Samples from West Bengal (0.417 μg/ml), Punjab (0.407 μg/ml) and Haryana (0.472 μg/ml) also showed good antiplasmodial activity in comparison to other *Aloe vera* samples. Extracts from Telangana (1.056 μg/ml) and Rajasthan (0.980 μg/ml) samples showed comparatively least antiplasmodial potential. The reported EC_50_ values of aloin and aloe-emodin were 67 μg/ml and 22 μg/ml respectively for antiplasmodial activity (Table 1). The value of positive control Chloroquine was 0.034 μg/ml. Antiplasmodial EC_50_ values and quantity of aloin and aloe-emodin in different samples has been shown in Fig. 3. Correlation between different parameters has been shown in Fig. 4. Both the tested phytochemicals i.e. aloin and aloe-emodin showed negative correlation with temperature and rainfall. But the effect was more pronounced on aloin concentration.

## Discussion

According to the WHO report (2016), there were an estimated 429,000 malaria deaths and 212 million new cases of malaria reported worldwide. This report revealed that 778,821 cases have been recorded with *P. falciparum* and 390,440 cases with *P. vivax* causing malarial infections in India. The WHO malaria report stated that African Region accounted for most global cases of malaria (90%), followed by the South-East Asia Region (7%) and the Eastern Mediterranean Region (2%) [5]. The 22% of India’s population live in high transmission, 67% live in low transmission areas and 11% live in malaria-free areas. The incidence of malaria in India accounted for 58% of cases in the South East Asia Region [33]. The central and eastern regions of India were reported for the most malaria cases particularly the eastern states of Odisha, West Bengal, and Jharkhand, the central states of Chhattisgarh and Madhya Pradesh, and the western states of Gujarat, Karnataka and Rajasthan, with the largest number of deaths reported in Odisha [34]. The diverse malaria epidemiology in India is mirrored by high diversity of malaria vector species, most of which exist as complexes comprising several cryptic species that vary in vectorial capacity [35, 36].

The climate varies from tropical monsoon in the south of the country to temperate in the north. Such climatic variation is due to a sharp temperature gradient caused by atmospheric changes in wind circulation and precipitation, lending to seasonally dependent asymmetric heating patterns of India’s peripheral bodies of water and land [3]. The new medicines are needed both to meet the challenge of malaria eradication and to circumvent resistance. The regions and periods of the year of plant collection are known to play an important role in the variation of the type of compounds found in plants as well as their concentration. Geographical conditions are the main factors for genetic diversity that ultimately affect the phytoconstituents and medicinal properties of a plant [37]. Diversity among organisms is a result of variations in DNA sequences and environmental effects. Species with a wide geographic area generally have more genetic diversity [38]. According to previous findings it is suggested that environmental temperature has a significant effect on antioxidant activity evaluation and it is more pronounced in cold weather [39]. This promoted us to collect samples in the winter season (Jan- Feb 2013).

*Aloe vera* grows all over the India and has a wide range of bio- active constituents found in leaves [40]. Nine categories of phytochemical constituents of *Aloe vera* can be classified as, anthraquinones*,* inorganic compounds, amino acids, fatty acids, alkaloids, carbohydrates, enzymes, and vitamins along with other miscellaneous compounds [41]. Anthraquinones are the most important active ingredients of *Aloe vera* [42]. The antiplasmodial activity of *A. vera* may be explained in the light of the presence of anthraquinones and other quinoid compounds which exert good activity against *P. falciparum* [43]. The four main anthraquinones showing quite high medical values are aloin, aloe-emodin, aloe bitter and aloe lectin [44].

Phytochemical extraction from plant samples greatly relies upon solvent and extract preparation methods [45, 46]. Water is the most polar of the solvents and confers negligible toxicity as such was used in this study [47, 48]. Standardization of natural products is a complex task due to their heterogeneous composition, which is in the form of whole plant, plant parts, or extracts obtained thereof. To ensure reproducible quality of herbal products, proper control of starting material is of utmost importance. For identification of the crude drug it is best to possess the authentic reference standard of that particular crude drug and HPTLC is a valuable tool for the investigation of herbal products with respect to different aspects of their quality and quantity [49, 50].

In present study, HPTLC analysis showed the variability in the amount of aloin and aloe-emodin anthraquinones contents with respect to diverse climatic conditions of Indian agro- climatic zones. The quantity of aloin is found higher in comparison to aloe-emodin. The previous studies also revealed the higher amount of aloin compare to aloe-emodin [51–53]. Maximum amount of aloin was detected in Jammu and Kashmir and Himachal Pradesh (Highland zone, with average temperature 13.5 °C and average rainfall 1011 mm and 1251 mm respectively) samples where as that of aloe-emodin in Punjab (Semi-arid zone, with average temperature of 25 °C and average rainfall 649 mm) sample.

There is not too much literature available on evaluation of antiplasmodial potential of *Aloe vera* but the plant has been reported several times as a antimalarial remedy in folk medicine system [25, 26]. Efforts are now being directed towards the discovery and development of new chemically diverse antimalarial agents. The South African *Vitex* spp. showed significant activity against *P. falciparum* chloroquine-resistant FCR-3, with IC_50_ values ranging from 9.16 ± 1.37 μg/ml to 16.02 ± 3.07 μg/ml. [54]. The plant extracts of 134 species tested for in vitro activity against a *Plasmodium falciparum* strain D10 using the parasite lactate dehydrogenase (pLDH) assay showed the 49% promising antiplasmodial activity (IC_50_ ≤ 10 g/ml), while 17% were found to be highly active (IC_50_ ≤ 5 g/ml) [55].

Larvicidal activity of *Aloe* species growing in Kenya, namely *Aloe turkanensis*, *Aloe ngongensis* and *Aloe fibrosa* tested against *A. gambie* third instar larvae showed 60% mortality at concentration of 2 mg/ml or 0.2% *w*/*v* [56]. Several anthraquinones extracted from different plant parts strongly inhibited in vitro growth of a chloroquine sensitive strain of *Plasmodium falciparum* (3D7) [57]. Anthraquinones may generate reactive oxygen and thus inactivate malaria parasites (*Plasmodium falciparum, P. vinckei and P. berghei*) [58, 59]. Latex leaf extract of *Aloe citrine* showed antiplasmodial activity due to presence of anthrone, homonataloin A/B as a major constituent [60]. On the basis of our present findings, Himachal Pradesh sample showed the lowest EC_50_ value (0.289) which means highest antiplasmodial activity in comparison to other *Aloe vera* samples. *Aloe vera* is a cold sensitive plant. During stress more phytochemicals are produced in plants to withstand the adverse conditions. Studies conducted on plants in stress conditions showed higher production of flavonoids, anthocyanins and mucilaginous substances [53].

In the present study EC_50_ values of aloin and aloe-emodin were 67 μg/ml and 22 μg/ml respectively for antiplasmodial activity against a chloroquine (CQ)-sensitive strain of *P. falciparum* (MRC-2). As both the anthraquinones tested in the study showed less antiplasmodial potential in comparison to crude extracts of different *Aloe vera* samples, these data suggest that the activity observed may be due to the synergistic effect of aloin, aloe-emodin and presence of other more active compounds in the extracts of *Aloe vera* plant. In contrast the previous study on Aloe extracts revealed that IC_50_ value for aloin was 169.76 ± 11.5 μg/ml against the chloroquine-sensitive 3D7 strain [27]. Dai et al. [61] reported the IC_50_ value for antiplasmodial activity of aloe-emodin was 50 μg/ml isolated from ethanol extract of South African plant *Kniphofia ensifolia* against *P. falciparum* Dd2 strain. They also showed that esterification of the primary hydroxyl group of aloe-emodin with various carboxylic acids increased its antiplasmodial activity, with the most potent analogue being the 3,4-dimethylcaffeic acid derivative with an IC_50_ value of 1.3 ± 0.2 μM) over 40 times than that of aloe-emodin. The biological activities of *Aloe vera* are due to the synergistic action of a variety of compounds, rather than from a single defined component [62, 63]. Significant correlation between quantities of both the anthraquinones used as marker compounds and EC_50_ values of the different *Aloe vera* extracts proves the plant as a prospective antimalarial remedy.

Traditional medicine and ethnobotanical information play an important role today as subject for scientific research, particularly when the literature and field work data have been properly evaluated. From the present work, it may be concluded that agro-climatic locations along with temperature and rainfall have significant effects on the *Aloe vera* plant phytoconstituents and its antimalarial potential. However, there is still a need to investigate the effects of different biotic and abiotic factors on *Aloe vera.* In future, it is also required to isolate antiplasmodial molecules from crude extracts of *Aloe vera* to enhance the antimalarial potential of the plant under cold stress. Plant growth and productivity are greatly affected by environmental stresses such as dehydration, high salinity, low temperature and biotic pathogen infection. Many plant genes are regulated in response to biotic and abiotic stresses and their gene products function in stress response. Such genetic systems are thought to be very important in increasing tolerance of plants to these stresses as well as in management for successful crop cultivation [64].

## Conclusion

Present work indicates that diverse climatic factors affect the quantity of aloin and aloe-emodin in different *Aloe vera* extracts, which plays a significant role in antiplasmodial potential of the plant. HPTLC analysis showed the variability in the amount of both the tested anthraquinone contents with respect to diverse climatic conditions of Indian agro-climatic zones. Significant correlation has been found between quantities of used marker compounds and EC_50_ values of the different *Aloe vera* plant samples. Study also concludes that north Indian *Aloe vera* samples are more potent against malaria parasite as compared to south Indian samples. These data lend support to the traditional use of *Aloe vera* in the treatment of malaria and further investigation would be worthwhile. Further studies to determine whether aloin and aloin-emodin do in fact act as synergistically and whether there are other antiplasmodial compounds present in the extract would also be valuable. Study also concludes that north Indian *Aloe vera* samples are more potent against malaria parasite as compared to south Indian samples. This study may help in selection, standardization and to formulate new and more potent antimalarial drugs of natural origin.

## Abbreviations

°C: Degree celcius
ACT: Artemisinin-based combination therapy
cm: Centi meter
CQ: Chloroquine
EC_50_: Effective concentration at 50%
GC: Gas Chromatography
HEPES: 2-[4-(2-hydroxyethyl) piperazin-1-yl] ethanesulfonic acid
HPLC: High Performance Liquid Chromatography
HPTLC: High Performance Thin Layer Chromatography
MIC: Minimum Inhibitory Concentration
mm: Milli meter
nLs^−1^: Nano liter per second
nm: Nano meter
pH: Hydrogen potential
R^2^: Regression Cofficient
RBC: Red blood corpuscles
R_f_: Retardation factor μg/ml
RPMI-1640 medium: Roswell Park Memorial Institute Medium
SPSS: Statistical Package for the Social Sciences
TLC: Thin layer chromatography
*v*/v/v: Volume by volume by volume
WHO: World Health Organization
